# A Case of Painless Elderly-Onset Rheumatoid Arthritis

**DOI:** 10.7759/cureus.61580

**Published:** 2024-06-03

**Authors:** Oliver Hayes, Sarkar Haider

**Affiliations:** 1 Geriatrics, Sherwood Forest Hospitals, Mansfield, GBR

**Keywords:** painless arthritis, antimicrobial stewardship practice, joint swelling, elderly onset rheumatoid arthritis, • rheumatoid arthritis

## Abstract

Rheumatoid arthritis (RA) has multiple manifestations. Patients present with a variety of symptoms and varying levels of severity. Elderly-onset rheumatoid arthritis (EORA) is described as RA with onset after 60 years of age. EORA can present with different clinical and laboratory findings compared to RA in a younger patient, making awareness of the condition important. Diagnosing inflammatory arthritis can be especially challenging in an elderly population where symptoms are poorly reported and communication is often difficult. We report the case of an elderly patient whose presentation with persistent tachycardia and raised inflammatory markers led to a diagnosis of EORA. This case details an atypical presentation of EORA with convincing diagnostic features for the disease without any joint symptoms reported. Clinicians should be aware of the differences in the typical presentation of EORA versus RA, the challenges of diagnosing inflammatory arthritis in elderly, isolated patients, and the importance of early diagnosis.

## Introduction

Rheumatoid arthritis (RA) is a complex inflammatory disease that can present diagnostic challenges to clinicians with its wide-ranging presentations and involvement of multiple body systems [1]. RA classically presents with pain, stiffness, and swelling in the joints of the hands, wrists, or feet over a period of many months [2]. RA can also affect the heart, lungs, kidneys, and eyes leading to diagnostic uncertainty and if left untreated, debilitating symptoms and increased mortality [3]. Constitutional features and blood tests of undiagnosed RA patients can mimic infection as well as malignancy, potentially causing misdiagnosis, over-investigation, and overtreatment with antibiotics [4,5]. Elderly-onset rheumatoid arthritis (EORA) is commonly defined as RA with an onset in patients over the age of 60 years. EORA patients are more likely to present with systemic features and higher inflammatory marker levels, resembling infection even more so than RA in younger patients [6]. Nevertheless, joint pain and swelling are prominent features in the majority of EORA patients [7]. Early diagnosis of RA and EORA is important to slow the progression of the disease, preserve function in affected joints, and control symptoms [8]. We report the case of an elderly woman presenting with predominant constitutional signs and an atypical absence of joint pain or tenderness. We discuss the contrast between the patient’s symptoms and laboratory findings and evaluate the contributing factors to the delays in her diagnosis.

## Case presentation

A female in her 70s was referred to the emergency department by community nurses concerned that the patient was confused and self-neglecting. The patient had a background of diverticular disease and severe hearing loss, communicating through written language and lip-reading. She used a stick to aid mobility but according to an accompanying friend, had not mobilized outside of the house for at least six months. The patient reported no symptoms. The patient was unkempt and thin, with the accompanying friend reporting unquantified weight loss. A shallow wound on the dorsum of the left foot with a small amount of discharge was noted on examination. Community nurses reported that the wound was the result of poor skin hygiene as well as abrasion from poorly fitting footwear. No other positive examination findings were noted at this time although no assessment of the patient's joints took place.

Vital signs on admission showed tachycardia of pulse rate of 120 beats/min, blood pressure of 160/76 mmHg, oxygen saturation of 96% on room air, respiratory rate of 18 breaths/min, and body temperature of 37.7℃. Laboratory blood tests showed raised inflammatory markers and mild hyponatremia (Table 1).

**Table 1:** Initial blood test results of the patient Blood tests showed raised inflammatory markers (WCC and CRP) as well as microcytic anemia, mild hyponatremia, and hypoalbuminemia.

Parameter	Level	Reference range
C-reactive protein (CRP)	128 mg/L	0-5 mg/L
White cell count (WCC)	11.3 x 10^9/L	4-10 x 10^9/L
Neutrophils	8.5 x 10^9/L	4-10 x 10^9/L
Lymphocytes	1.0 x 10^9/L	1-3.0 x 10^9/L
Platelets	386 x 10^9/L	150-410 x 10^9/L
Hemoglobin	100 g/L	120-150 g/L
Mean corpuscular volume	82 fL	83-101 fL
Serum sodium	129 mmol/L	133-146 mmol/L
Serum potassium	4.5 mmol/L	3.5-5.3 mmol/L
Urea	2.9 mmol/L	2.5-7.8 mmol/L
Creatinine	30 mmol/L	55-100 mmol/L
Bilirubin	6 umol/L	0-21 umol/L
Alanine aminotransferase	8 U/L	0-35 U/L
Alkaline phosphatase	99 U/L	30-130 U/L
Albumin	20 g/L	35-50 g/L
International normalized ratio (INR)	1.2 ratio	Ratio

Blood and urine cultures showed no growth and chest X-ray showed no abnormalities. A swab from the patient's foot wound grew no pathogens. ECG showed sinus tachycardia with normal PR interval and normal corrected QT interval. The patient was admitted to a general medical ward for regular blood monitoring and to receive broad-spectrum IV antibiotics for an infected foot wound. A further low-grade fever of 37.8℃ was noted on the second day of admission. The patient was noted to have continuous confusion since admission and following a CT head scan and collateral history from relatives, was diagnosed with vascular dementia by the hospital psychiatric team.

After receiving a course of antibiotics, the patient was deemed medically optimized for discharge and was transferred to a physical rehabilitation ward at a community hospital for physiotherapy input. On review at the community hospital, the patient was noted to remain tachycardic at 112 beats/min and repeated blood tests showed no significant improvement in inflammatory markers. C-reactive protein (CRP) had minimally improved from 128 mg/L to 120 mg/L and white cell count (WCC) had increased from 10.5 x 10^9/L to 12.0 x 10^9/L. Repeat ECG again showed sinus tachycardia. Urine culture remained negative. Low-grade pyrexia of 37.8℃ was again observed.

The patient was given a course of oral antibiotics for untreated foot wound infection. This had no beneficial effect on pulse rate or inflammatory markers. A CT of the thorax, abdomen, and pelvis was performed and showed no convincing sinister pathology to explain the patient’s clinical picture. There was an incidental finding of an old L1 vertebral fracture only, which was managed conservatively. A more detailed clinical examination found the patient had swellings of the metacarpophalangeal joints bilaterally although these were non-tender on palpation and the patient repeatedly denied any pain in the hands. There were no other notable features on assessment of the hands or feet and examination of large joints showed no abnormalities. A collateral history from the patient’s friend established at least a six-month history of intermittent joint swelling in the hands which was never clinically assessed.

Further blood tests were performed showing very high levels of rheumatoid factor, erythrocyte sedimentation rate, and cyclic citrullinated peptide antibody (Table 2).

**Table 2:** Further blood results showing raised ESR, rheumatoid factor, and CCP antibody levels Very high levels of ESR, rheumatoid factor, and CCP antibodies suggesting a strong possibility of rheumatoid arthritis. ESR: erythrocyte sedimentation rate; CCP: cyclic citrullinated peptide

Parameter	Level	Reference range
Erythrocyte sedimentation rate (ESR)	110 mm/1hr	0-35 mm/1hr
Rheumatoid factor	>650 kU/L	0-14 kU/L
Cyclic citrullinated peptide (CCP) antibody	>340 IU/mL	<7 IU/mL

**Figure 1:**
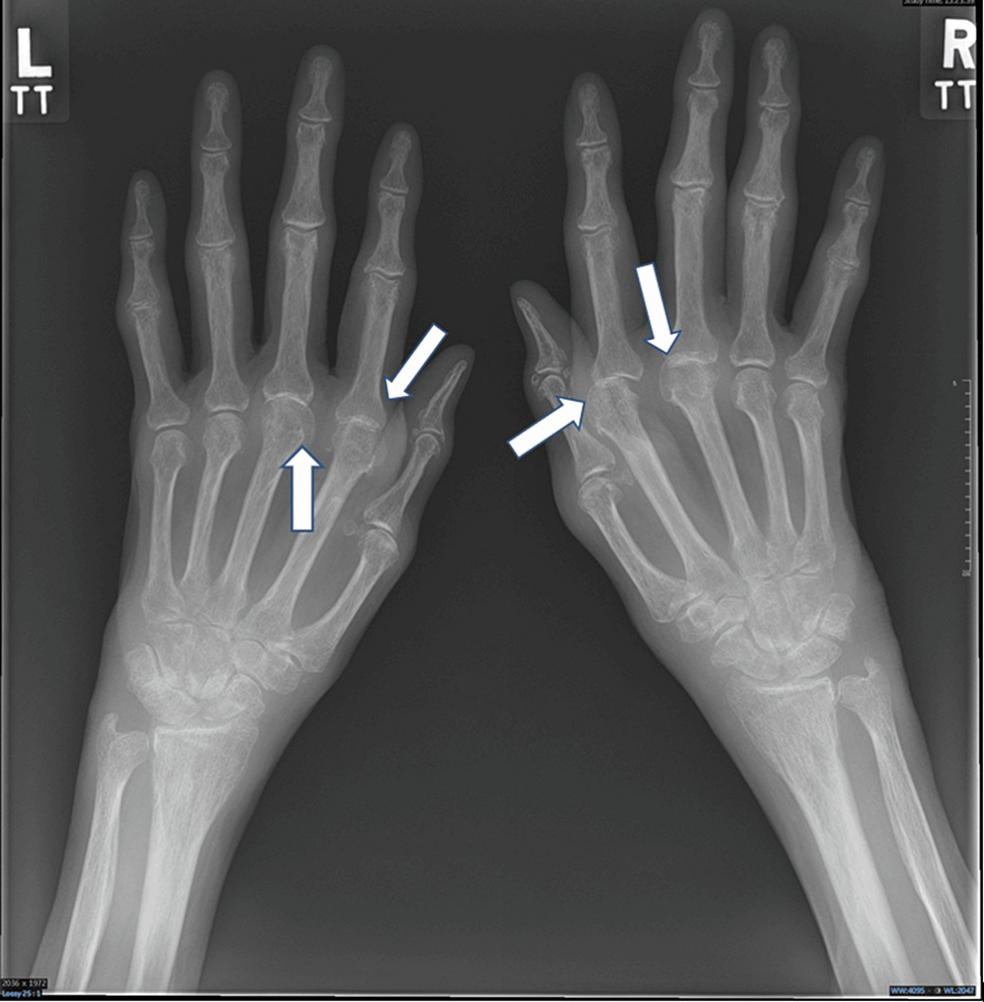
Hand X-ray showing subluxation of the second and third metacarpophalangeal joints bilaterally White arrows pointing toward affected joints.

Hand X-ray showed subluxation of the second and third metacarpophalangeal joints bilaterally (Figure 1).

The patient was reviewed by a rheumatologist who confirmed the diagnosis of RA based on the clinical findings and blood results above. The patient was treated initially with a weaning course of prednisolone. Inflammatory markers and pulse rate improved following the initiation of steroids and the patient was transferred back to the same rehabilitation ward. The rheumatology team plans to see the patient as an outpatient to consider initiation of disease-modifying antirheumatic drugs. An echocardiogram has been requested as an outpatient to further investigate the patient's tachycardia.

## Discussion

This case involved a patient with RA with very high laboratory markers for RA. X-ray findings were suggestive of RA, but unusually, no symptoms to report and no tenderness of the affected joints. Joint pain affects the daily lives of the majority of patients with RA, even those established on treatment [9]. Cases of painless synovitis in RA are rare but have been reported and have been shown to follow a similar disease progression to those with pain [10]. This report demonstrates a case of RA with a complete lack of pain as well as entirely non-tender joints on palpation. This combination of clinical findings is very atypical, especially considering the patient’s blood tests would suggest a highly active disease [11]. This contributed to a delay in diagnosis and also demonstrates the value of a thorough examination when diagnosis is unclear, even when there is limited history to guide that assessment.

Our case displayed many of the typical features of EORA. Clinically, EORA has a tendency to present more acutely with prominent weight loss and lethargy compared to younger-onset RA [6]. On laboratory tests, EORA patients generally have higher CCP antibody titers as well as high CRP, WCC, and erythrocyte sedimentation rate (ESR) levels as was seen in this case [7]. Although not present in our case, large joints such as shoulders and knees are more likely to be affected in EORA compared to younger-onset RA [12].

The only clinical signs that our patient exhibited were subjective weight loss, low-grade fever, and tachycardia. Tachycardia is common in patients with RA due to generalized inflammation, pericardial involvement (in up to 50% of patients), or ventricular dysfunction [13]. Tachycardia and low-grade fevers are features shared with infection and can contribute to misdiagnosis [14]. It was not recognized that despite a course of antibiotics and improvement in the patient’s foot wound, pulse rate remained high and no improvement was seen in inflammatory markers.

The patient may have been suffering from RA for an extended period of time but did not seek medical attention, likely due to cognitive impairment and isolation. This highlights how elderly and socially isolated patients are at risk of having significant medical diagnoses missed, which can lead to poor health outcomes [15]. Reciprocally, RA and arthritis more generally can be a major cause of impaired mobility and function as seen with the patient in this case report. This physical limitation can contribute to withdrawal from society and low mood [16].

## Conclusions

This case highlights to clinicians the need to consider a diagnosis of EORA in elderly patients presenting to acute hospitals with vague symptoms and emphasizes the need to adapt diagnostic focus when initial management has no effect on a patient’s clinical condition. Our case involved a patient with an atypical lack of symptoms despite significantly deranged blood tests. This underlines the varied nature of RA presentations and the need to examine patients extensively when there is diagnostic uncertainty. The numerous physical and mental health complications associated with RA, especially if left untreated, make swift diagnosis essential.
